# Terrestrial LiDAR point cloud dataset of cocoa trees grown in agroforestry systems in Cameroon

**DOI:** 10.1016/j.dib.2024.110108

**Published:** 2024-01-29

**Authors:** Emilie Peynaud, Stéphane Momo Takoudjou

**Affiliations:** a CIRAD, UMR AMAP, F-34398 Montpellier, France. AMAP, Univ Montpellier, CNRS, CIRAD, INRAE, IRD, Montpellier, France; b Plant Systematics and Ecology Laboratory, Higher Teachers' Training College, University of Yaoundé I, P.O. Box 047 Yaoundé, Cameroon; c Gembloux Agro-Bio Tech, TERRA Teaching and Research Centre, Forest is Life, University of Liège, Gembloux, Belgium

**Keywords:** LiDAR, Theobroma, Leaf/wood segmentation, Tree architecture, Biomass, Agroecological forestry

## Abstract

This paper presents a dataset aimed at characterizing cocoa trees cultivated within complex agroforestry systems managed by smallholder farmers in the Central region of Cameroon. The dataset highlights the architectural structure of the trees as well as the distribution of their leaves and wood using 3D point clouds obtained through the Leica ScanStation C10 terrestrial LiDAR. The data collection campaign was conducted in August 2019 in the district of Bokito (latitude 4°34′ N and longitude 11°07′ E), specifically within the village of Yorro located in a transition zone between forest and savannah. The dataset includes information on 55 cocoa trees, spread over five distinct architectural types. These trees were sampled from various age stands ranging from 5- to 70-year-old. For 29 of these trees, a leaf/wood segmentation of the point clouds was performed. For each of these trees, the dataset comprises the raw point cloud of the entire tree, as well as separate point clouds for the leaves and wood, each in two distinct sets of 3D points. The data provides the foundation for conducting numerous cocoa tree measurements based on their representation in point clouds, allowing for a more comprehensive understanding of their architecture, photosynthetic capacity, and distribution of above-ground biomass.

Specifications Table

**Table:** 

Subject	Plant Science: General, Ecological Modelling, Biostatistics
Specific subject area	Characterisation of complex cocoa-based agroforestry systems
Data format	Raw, Analyzed
Type of data	Table
Data collection	Terrestrial LiDAR point clouds of cocoa trees were collected using a Leica ScanStation C10. Trees were sampled in various agroforestry plots representative of different stand development phases and managed by farmers in Bokito district. The age of the stands ranged in age from 5 to 70 years, age being the time elapsed since the creation of the cocoa stand. The trees sampled are classified into different architectural types [1]. A leaf/wood segmentation was performed with LeWoS algorithm [2]. Point clouds of trees exhibiting poor quality due to occlusion by surrounding vegetation were discarded from the original dataset.
Data source location	City: Yorro, Bokito district in Central Cameroon Country: Cameroon Latitude and longitude: 4°34′ N and 11°07′ E
Data accessibility	Repository name: Dataverse Cirad Data identification number: 10.18167/DVN1/5HZB1F Direct URL to data: https://doi.org/10.18167/DVN1/5HZB1F Instructions for accessing these data: scroll down to the “Files” tab that shows a list of the three files composing the repository. Data files must be downloaded one by one since their size exceed the limit of 100 MB. Select one file, then click on the “Access File” icon and on “ZIP Archive” or directly click on the “Download” button. Accept the Dataset Terms by clicking on the “Accept” button to start downloading.

## 1 Value of the Data

• The dataset is useful to characterize, along time sequences, the architecture and biomass of cocoa trees cultivated within agroforests in Central Cameroon. These cocoa-based agroforests are sustainable and resilient toward climate change and socio-economic context. Understanding their functioning as ecosystems and as cropping systems is a crucial key that may help to design sustainable management practices for plants to address issues like biomass and biodiversity conservation and food security.
• The data are valuable for scientists, researchers and engineers interested in the characterization and modeling of cocoa trees cultivated in agroforestry systems. Data and computer scientists working on segmentation algorithms can also benefit from these data: the point clouds are segmented into leaf and wood parts so that they can be used as a reference for training or comparison of algorithms.
• This dataset consists of terrestrial LiDAR point clouds that provide in silico representations of plants from which one can carry out many non-destructive measurements. For example, these measurements encompass the volume of wood, the branch diameter, order and angle distribution, and the 3D-distribution of foliage according to the age of the stand or according to the tree architectural type or any other architectural metrics of interest [3].
• To exploit the dataset, there are softwares and algorithms specifically designed to analyze terrestrial LiDAR data for vegetation. The QSM algorithm [4] combined with the AmapStudioScan [5,6] software compute estimates of the volume of wood by automatically fitting cylinders along the trunk and branches. The AmapVox software [[7], [8]] can estimate metrics such as the leaf area density (LAD) or the plant area index (PAI) using user informed parameters like the angular distribution of the leaves. These are examples of tools that can help to perform accurate measurements of the trees from the dataset.
• Architectural types can be difficult to distinguish in the field. The dataset can be used to characterize the architectural types of the cocoa trees with respect to traits like wood volume, wood surface, number of axes, branching structures, ramification types, diameter and length of branch… This study could help to identify traits that are easy to evaluate or to measure in the field, specific to each type, which would enable them to be seamlessly identified.
• The dataset provides an overview of the cocoa trees over the long term, as the trees belong to plots of age ranging from 5- to 70-year-old. In addition, the succession of architectural types provides information both about the vegetative states of the trees and on the results of management practices implemented by farmers in the long term to rehabilitate and rejuvenate agroforests.
• Retrospective analysis of cocoa tree architecture could possibly be done thanks to the dataset, provided that all bottlenecks associated with transcribing LiDAR data into architectural information are overcome. Combined with additional studies on anatomy and yield, this would make it possible to study the effects of pruning on the tree growth and development, for example. It would also enable the development of tools to better assess tree vigor and health.

## 2 Data Description

**Fig. 1:**
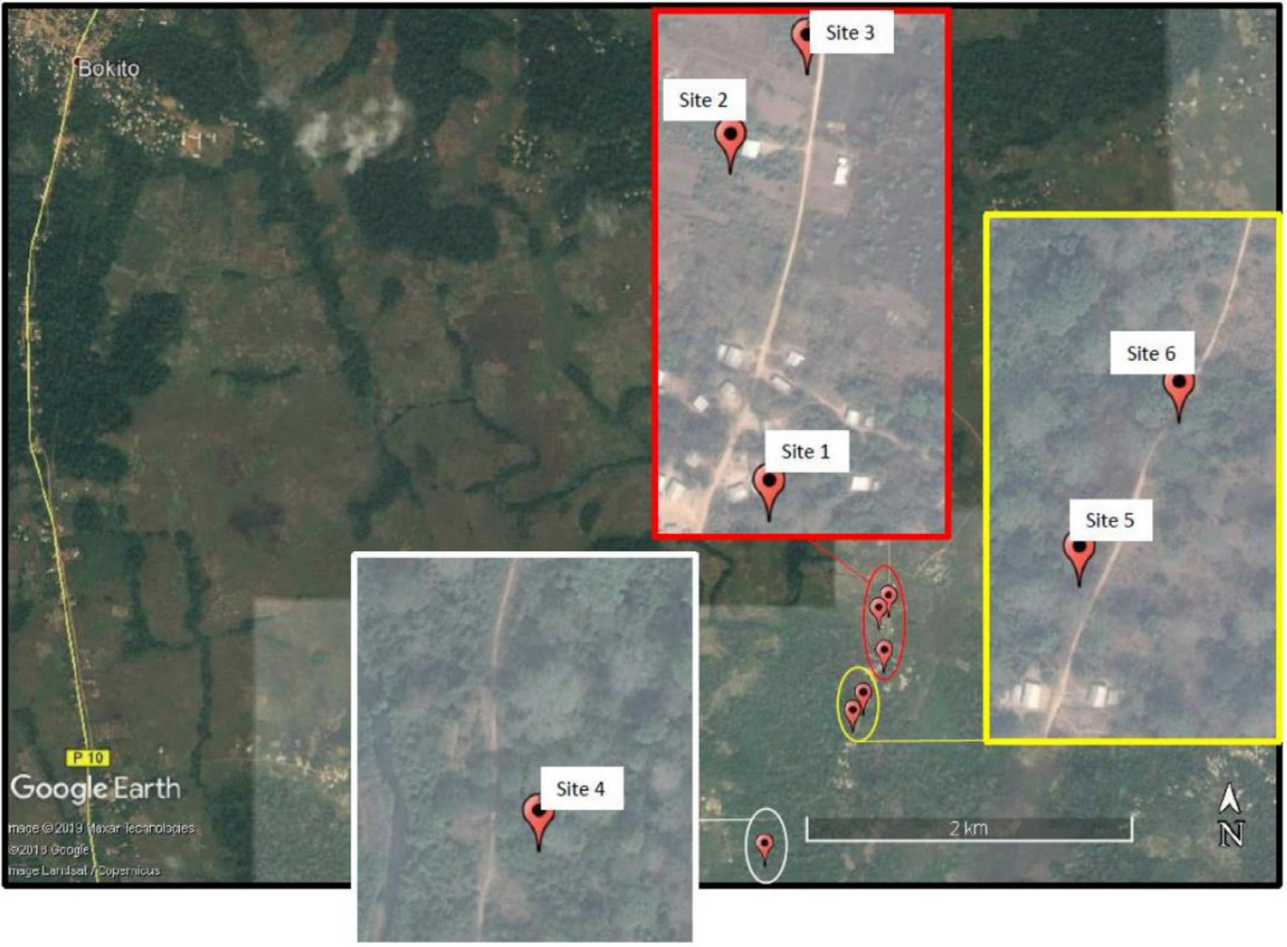
Satellite view of different sampling units in Yorro village where the LiDAR data have been taken. Remark: the data collected at sampling unit 5 was discarded from the dataset. Credit: Stéphane Momo Takoudjou.

The dataset [9] consists of a collection of point clouds representing cocoa trees grown in complex agroforestry systems. A point cloud is a set of discrete points in 3D space characterized by their Cartesian coordinates stored as tabular data in space separated value text files. The dataset was built up from LiDAR scans performed from the 27th to the 30th of August 2019 in the district of Bokito in the village of Yorro located in a forest-savannah transition zone in Central Cameroon. The dataset gathers 3D scans of trees sampled on different sampling units shown on Fig. 1 where Bokito is in the upper left corner. These sampling units are cocoa-based agroforestry systems traditional to Central Cameroon and managed by farmers according to low-input practices. Farmers harvest cocoa pods when they reach ripeness: some trees still have fruits (not ripe yet) and some have no fruit because they have already been harvested or they are not productive yet. Table 1 gives the age of each stand and its unit identification as it appears in the database and its unit number according to Fig. 1. Sampling unit 5 does not appear in this table because scans acquired at this site were discarded from the original dataset. The cocoa trees of the dataset belong to 5 architectural types according to the classification of [1]. The three first types are representative to the vegetative growth of the cocoa trees. Types 3 and 4 are the results of the management practices performed by farmers on the long term. Table 1 also gives for each site the number of trees per architectural type in the dataset.

**Table 1:** Sampling unit identification labels as they appear in the dataset, sampling unit numbers with respect to Fig. 1, ages of the stands and numbers of trees per architectural type.

Sampling unit label	Sampling unit number	Age of the stand	Number of trees for each type						Total
			T0	T1	T2	T3	T4	T5	
Obama	Site 1	5	1	7	4	1	0	0	13
Oloumou	Site 2 et 3	10	7	2	3	5	0	0	17
Christ	Site 4	20	0	3	3	2	5	0	13
Alino	Site 6	70	0	3	3	3	3	0	12

The dataset [9] is stored in the linked repository composed as follows:

• full-cocoa-trees.zip is an archive containing ASCII files in which are stored the point clouds of 55 cacao trees. Each file contains the list of the 3D-coordinates (x,y,z) and the returned laser signal intensity of each point of a single tree.
• leaf-wood-segmented-cocoa-trees.zip is also an archive containing the point clouds of the trees that have been segmented into leafy and woody parts. For each of the 29 segmented trees, there are 3 files: one file for the point cloud of the whole tree, one file for the point cloud of the leafy part and one file for the point cloud of the woody part.
• readme.txt is a file that briefly summarizes the contents of the dataset. It gives the key that explains how the names of the ASCII files were constructed. This key identifies the site from which the scanned trees originate (sampling unit label on Table 1) and their architectural types. The readme.txt file also gives GPS coordinates of the sampling units.

## 3 Experimental Design, Materials and Methods

### 3.1 Data acquisition in the field

**Fig. 2:**
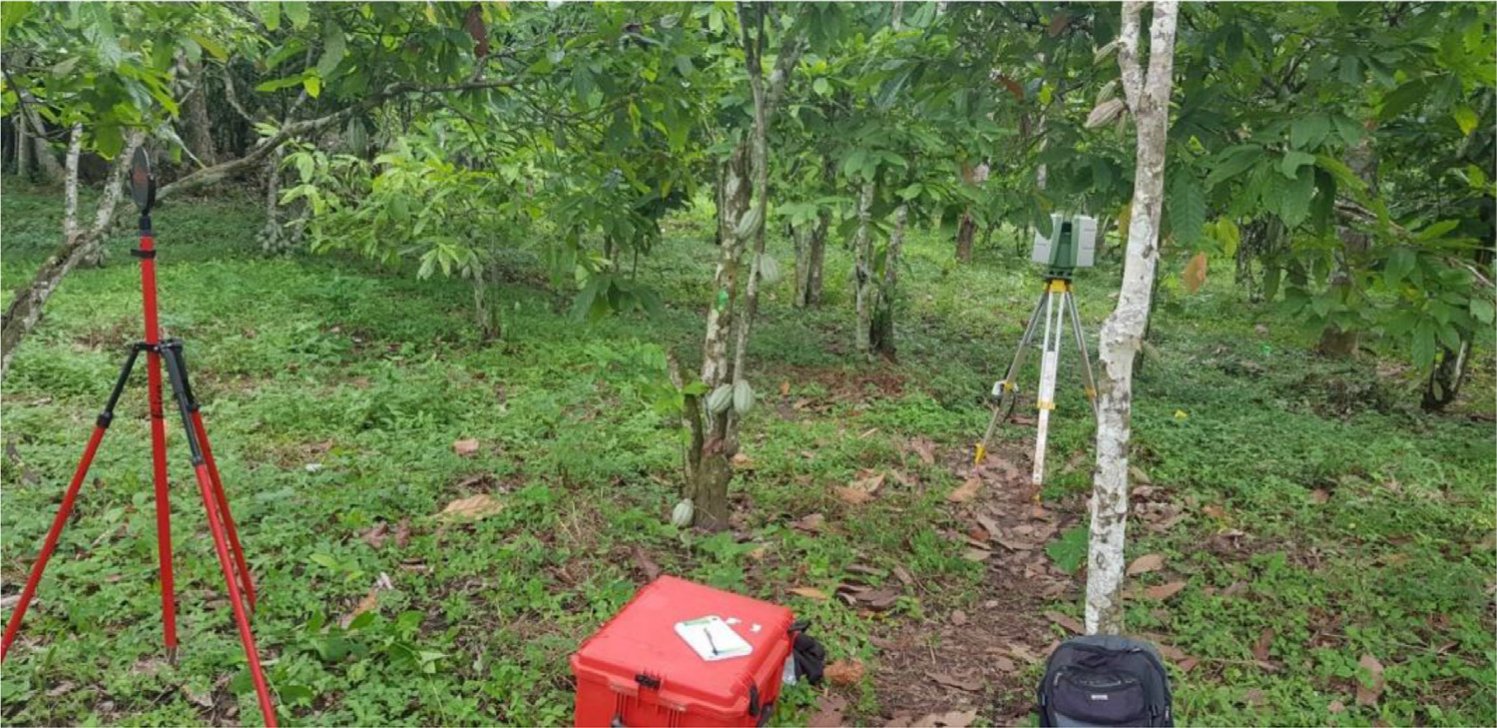
Scanning a cocoa based agroforestry system in Cameroon. The scanner is in the background (in grey and green). One of the spherical targets used to adjust the successive scan positions is in the left foreground. Credit: Stéphane Momo Takoudjou.

**Fig. 3:**
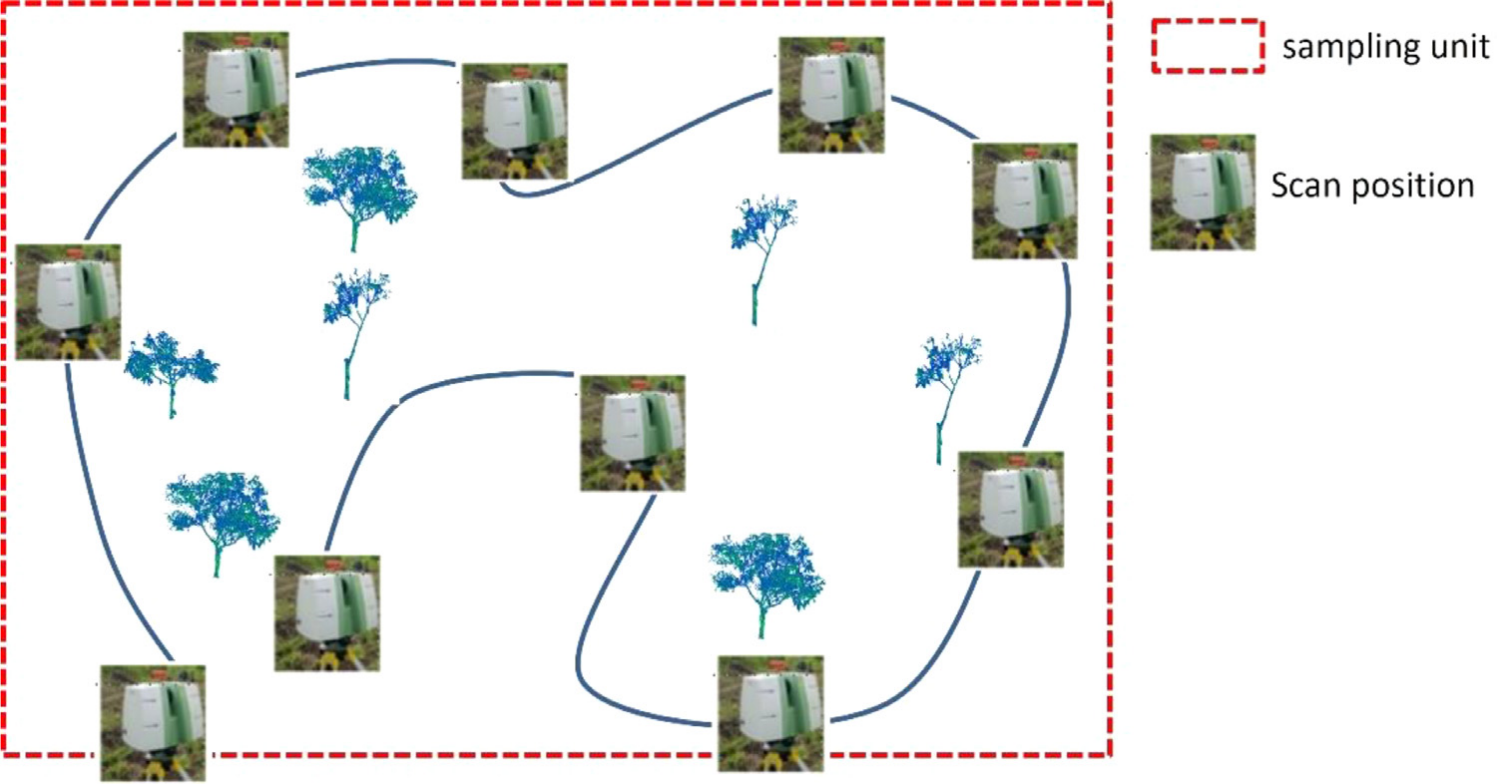
The scanning positions in a plot according to the pathway method. Credit: Stéphane Momo Takoudjou.

**Fig. 4:**
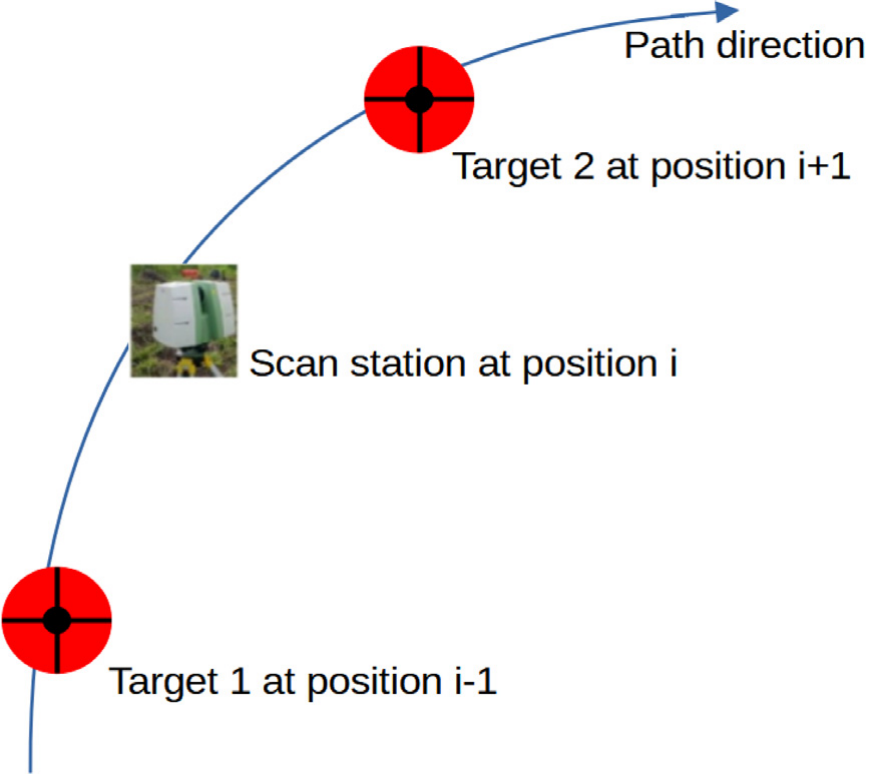
Configuration of the scan station and the two targets for one position of the pathway. The pathway method consists in scanning the trees from different positions. The successive positions form a path through the plot. Target 1 and 2 materialize respectively the previous and the next scan station positions. Credit: Emilie Peynaud.

The terrestrial laser scan data acquisitions were performed thanks to a terrestrial LiDAR scanner (TLS), ScanStation C10 (Fig. 2). Since trees are 3D objects, they were scanned more than one time from independent locations. These locations were positioned to enable construction of 3D point clouds, through alignment of overlapping point clouds and to minimize occlusion (data hidden from the scanner). On each sampling unit, we scanned the cocoa trees of interest from various positions according to the pathway method (see [10] for more information) . The successive positions follow a path that optimizes the visibility of the cocoa trees of interest (Fig. 3). At each position, two spherical red targets are used to indicate respectively the previous and the next scan positions (Fig. 4). These targets materialize specific overlapping points that are used as a reference to align the point clouds recorded at each scan position of the path. It forms a raw point cloud showing a portion of the plot observed from the path. The scanning resolution was set to a spacing of 0.05 m between points at 100 m. The Leica company suggested this pathway method and scanning parameters to co-register multiple scans in the same coordinate system.

### 3.2 Data processing

**Fig. 5:**
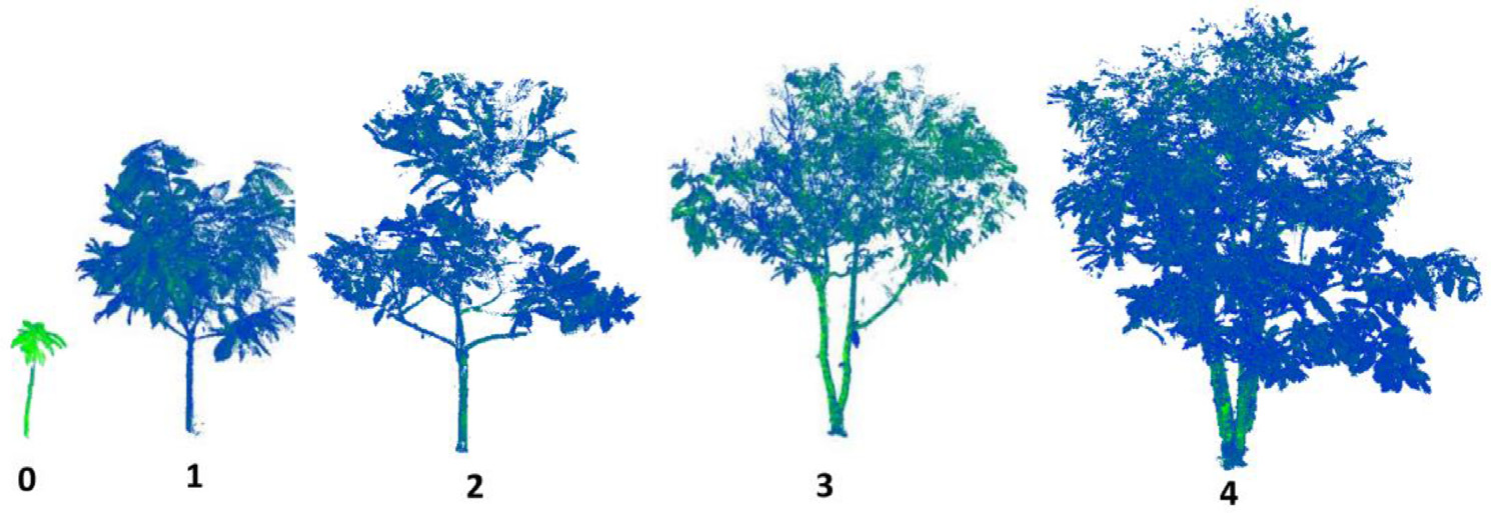
Raw point clouds of cocoa trees of types 0 to 4 according to the classification of [1]. Credit: Stéphane Momo Takoudjou.

**Fig. 6:**
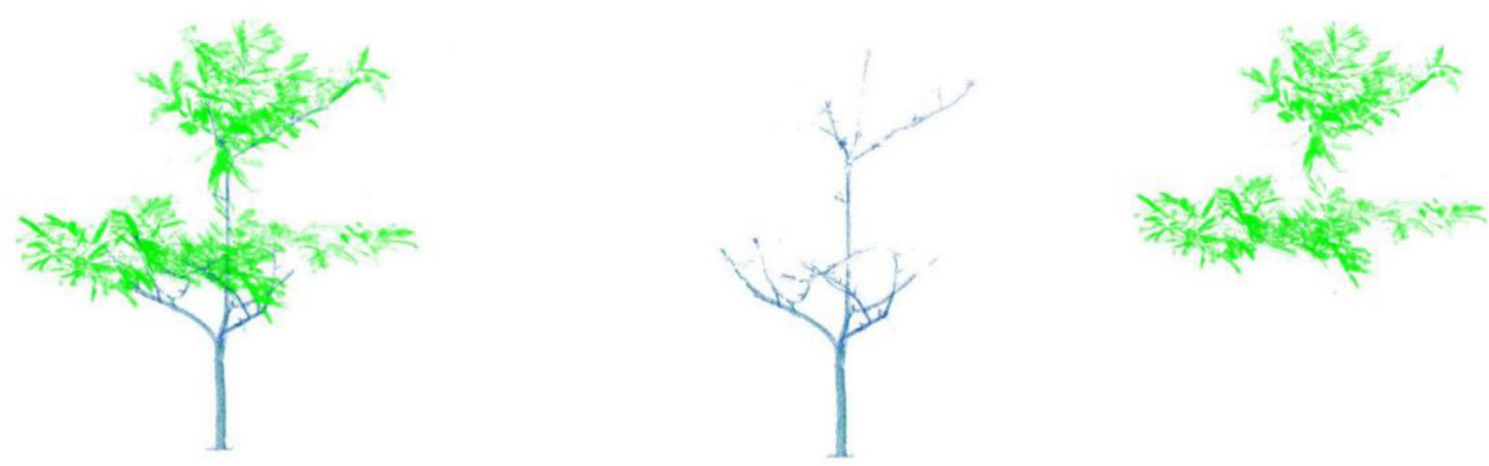
Leaf/wood segmentation. From left to right: raw point cloud of an entire coco tree, point cloud of the woody part of the tree, point cloud of the leafy part of the tree. Credit: Stéphane Momo Takoudjou.

The co-registration of the point clouds from the different scanning positions was performed using the Leica Cyclone software *version 9.4.2*. This produces a raw 3D points cloud showing the trees and the surrounding environment (the soil, grass, neighboring trees). A point is determined by its x, y and z-coordinates and it is characterized by the intensity of the laser ray transmitted to the scanner after it has hit the point. Then, the trees of interest were extracted from the raw point cloud using the 3DForest software *version 0.5* [11]. The trees are stored in the dataset as tabular data consisting in the Cartesian coordinates and laser intensity and stored in text files. The point clouds can be visualized by opening those text files with software such as CloudCompare [12]. Fig. 5 shows examples of point clouds of five cocoa trees of type 0 to 4 viewed in CloudCompare. We discarded from the dataset the trees of low point cloud quality (high level of occlusion by surrounding vegetation). Then, for the good-quality point clouds, we performed the segmentation of the leaf and wood points thanks to the LeWoS software [2]. An example of the leaf and wood segmentation is shown on Fig. 6. The raw point clouds from which we extracted the trees are not included in the dataset for reasons of memory capacity and clarity of purpose, but they may be the subject of a future repository allowing access to large files to provide insight on cocoa agroforests at the sub-plot scale.

## Limitations

Apart from the fact the LiDAR device is heavy to transport, and the scanning time required for a good resolution is long (30 min per scan), the main difficulties are related to the fact that the object should not move during the data acquisition. Leaves and small branches moving due to the wind can cause inaccurate and blurred point clouds. In addition, the structure of an agroforest is complex; many scan positions must be taken to reduce occlusion by the surrounding vegetation: the trees of interest must be scanned from various viewpoints to ensure a decent quality of 3D point clouds. In some areas where the canopy is closed, the upper part of the trees may suffer from low resolution due to the fact that the scans were performed from the ground. Closed canopy of cocoa trees may also lead to inaccuracies in the extraction of individual trees from raw point clouds. Finally, there is a trade-off between the resolution, accuracy of the 3D point cloud, the acquisition time, and the size of the data.

The sampling of the cocoa trees is not rigorously representative of the distribution of architectural types in a plot. For example, type 4 which corresponds to rejuvenated trees, tends to be more frequent in old plots than in new ones and is the most frequent type in old plots (see [1]). Plots were selected if they belonged to farmers who agreed to give us access to the plots and if the plots were bordered by drivable roads to facilitate transport of the apparatus, which may introduce an additional sampling bias.

The cocoa-based agroforests in the region of Bokito are composed by Amelonado and hybrids of Forastero introduced over the last century. The genetic material of the cocoa trees of the dataset is not precisely defined, since the agroforest are composed by cocoa trees from different sources. For example, farmers replace dead cocoa trees by seedlings obtained from nurseries or by direct sowing of beans harvested from productive and resistant trees.

The dataset shows some cocoa trees with fruits, while some have no fruit at all. Some trees are not yet productive, while others have been harvested and others have not at the time of the data acquisition, since farmers wait until the ripeness before harvesting the pods. For these reasons, the dataset does not give exhaustive information on tree productivity. Additional data acquisitions should be done to better characterize the yield. For example, establishing or assessing allometric equations to predict the number or the volume of pods per plants before harvest could be of great benefit for cocoa producers.

## Ethics Statement

The acquisition of the data was approved by all farmers and was conducted in agreement with them. The authors have read and follow the ethical requirements for publication in Data in Brief and confirming that the current work does not involve human subjects, animal experiments, or any data collected from social media platforms.

## CRediT Author Statement

**Emilie Peynaud**: Writing- original draft preparation, reviewing and editing, funding acquisition. **Stéphane Momo Takoudjou**: Methodology, Investigation, processing data, Writing- Reviewing and Editing.

## Data Availability

Cocoa tree point clouds obtained by terrestrial LiDAR scanning in agroforestry systems in Cameroon (Original data) (Dataverse).
